# Homoterpene Biosynthesis in Fungi

**DOI:** 10.1002/anie.202517837

**Published:** 2025-11-17

**Authors:** Lin Zhou, Tatjana Reuter, Kiara Schumann, Markus Mayer, Dominik Matthias Hanauska, Lena Barra

**Affiliations:** ^1^ Department of Chemistry University of Konstanz Universitätsstraße 10 78464 Konstanz Germany

**Keywords:** Apple canker, Enzyme discovery, Homoterpenes, Isoprene rule, Non‐canonical terpenoids

## Abstract

Homoterpenes carrying an additional methyl group in their carbon backbones are an emerging class of natural products that challenge the biogenic isoprene rule, stating that terpenes are composed of integer multiples of C_5_ units. We and others have recently shown that biosynthetic pathways to homoterpenes are widespread in bacteria, leading either to specialized scaffolds such as the “Greek philosophers homoterpenes” in Pseudomonadota or to simple methyl analogs of central eudesmanes and germacranes (“humanists homoterpenes”) in Actinomycetota. Here we report the discovery of the first homoterpene biosynthetic pathway in the fungal kingdom using targeted genomic data mining in combination with in vitro pathway reconstitution. Functional analyses of a fungal methyltransferase (NdiMT) and terpene cyclase (NdiTC) pair from the plant‐pathogenic fungus *Neonectria ditissima*, the causative agent of apple canker, led to the discovery of a novel homosesquiterpene featuring an intriguing heptamethylbicyclo[3.3.1]nonane scaffold. Phylogenetic analyses indicate that the fungus acquired the key methyltransferase via horizontal gene transfer from bacteria, whereas the terpene cyclase appears to have evolved from a fungal ancestor. The discovery raises fundamental questions about the evolutionary rationale and functional consequences of terpene methylation in nature.

## Graphical abstract

The first homoterpene biosynthetic pathway in the fungal kingdom is reported. A methyltransferase–terpene cyclase pair produces a unique heptamethyl bicyclo[3.3.1]nonane homoterpene via presodorifen, demonstrating that homoterpene biosynthesis via the addition of a C_1_ unit to terpene diphosphate precursors prior to scaffold formation extends beyond the bacterial domain to eukaryotes.

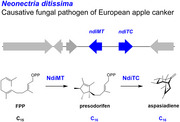

Terpenes constitute the largest class of natural products and hold substantial value as medicinal drugs,^[^ ^1^, ^2^, ^3^, ^4^ ^]^ biofuels,^[^ ^5^, ^6^ ^]^ and aroma or fragrance compounds.^[^ ^7^, ^8^ ^]^ In an ecological context, volatile terpenes often mediate important intra‐ and interspecies communication, acting as signaling compounds.^[^ ^9^, ^10^, ^11^, ^12^ ^]^ Terpenes are also commonly referred to as polyisoprenoids, since their carbon skeletons are typically composed of integer multiples of C_5_ units. This central dogma was recognized as early as 1953 by Ružička^[^ ^13^ ^]^ and is known as the “biogenic isoprene rule,” which reflects their shared biosynthetic origin from the universal C_5_ building blocks dimethylallyl diphosphate (DMAPP) and isopentenyl diphosphate (IPP). DMAPP and IPP are condensed by prenyltransferases to yield oligoprenyl diphosphates such as geranyl diphosphate (GPP, C_10_), farnesyl diphosphate (FPP, C_15_), and geranylgeranyl diphosphate (GGPP, C_20_). These acyclic intermediates are subsequently converted by terpene cyclases (TCs), which construct the typically complex (poly)cyclic backbones frequently observed in terpene natural products.^[^ ^14^, ^15^, ^16^ ^]^

Homoterpenes, which carry an additional C_1_ unit in their carbon backbones and hence deviate from the biogenic isoprene rule, have long been regarded as exceedingly rare and exotic in nature. In the kingdom of plants, one biochemical pathway to the acyclic homoterpenes 4,8‐dimethyl‐1,3,7‐nonatriene (DMNT, **1**, C_11_) and 4,8,12‐trimethyltrideca‐1,3,7,11‐tetraene (TMTT, C_16_) has been characterized. Both are derived via the oxidative removal of a C_4_ unit from (*E*)‐nerolidol (**2**, C_15_→C_11_) and (*E*,*E*)‐geranyllinalool (C_20_→C_16_), respectively, which is mediated by a cytochrome P450 monooxygenase (Figure 1a). DMNT and TMTT are important signaling compounds that contribute to the chemical defense of the producing plant against herbivorous insects.^[^ ^17^, ^18^ ^]^

**Figure 1:**
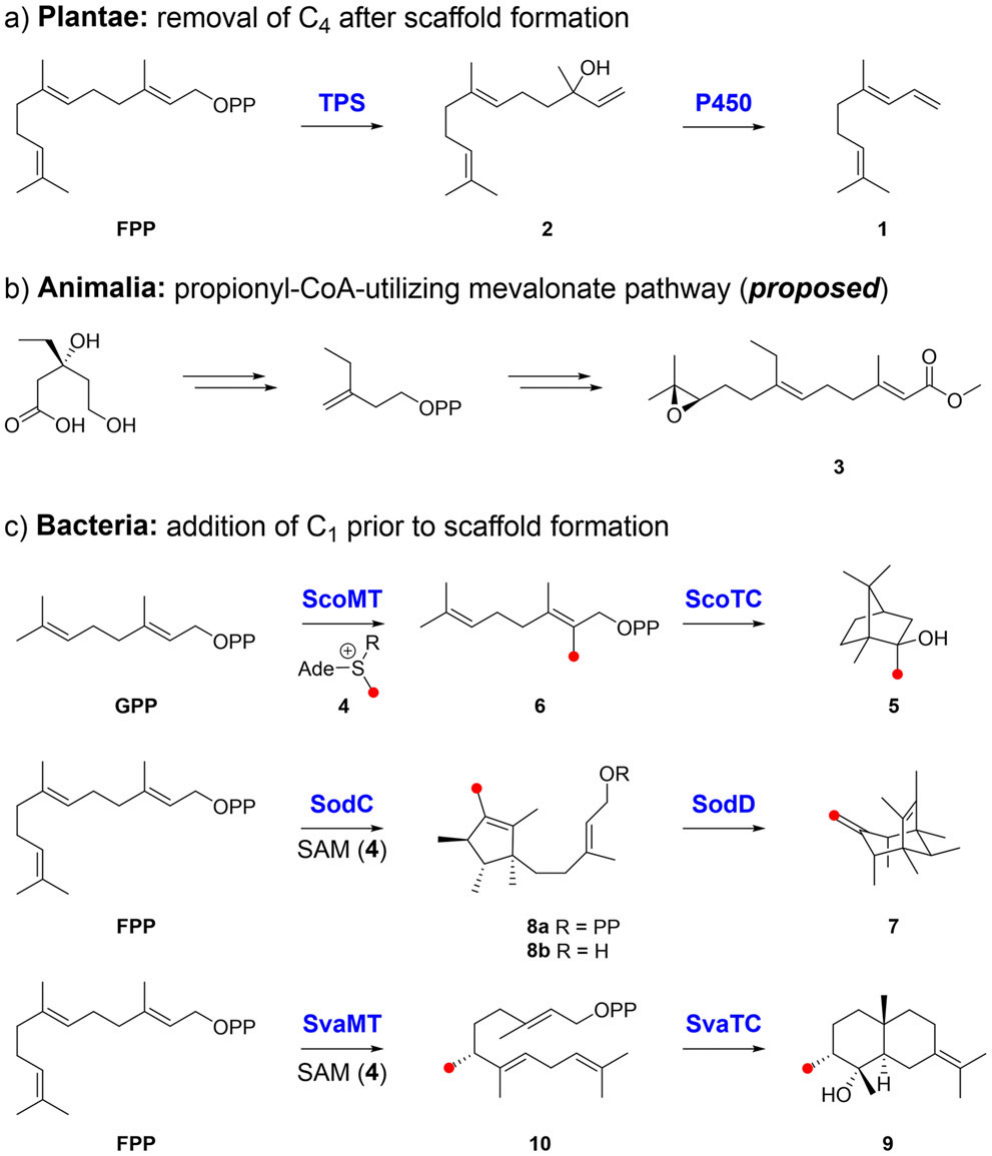
Reported homoterpene biosynthetic pathways. a) Pathway to DMNT (**1**) in plants. b) Proposed pathway to juvenile hormone II (**3**) in insect *Manduca sexta*. c) Pathway to 2‐methylisoborneol (**5**), sodorifen (**7**), and kantenol (**9**) in bacteria. Ade  =  adenosyl; R = 1‐aminocarboxypropyl; TPS = terpene synthase.

In the kingdom of animalia, it has been proposed that juvenile hormone II (**3**) from the insect *Manduca sexta* is biosynthesized via an alternative mevalonate pathway that utilizes propionyl‐CoA instead of acetyl‐CoA as a building block (Figure 1b). However, besides feeding experiments with isotopically labeled propionate, no biochemical evidence has been reported.^[^ ^19^ ^]^ In bacteria, a third biosynthetic strategy has been characterized that entails a methyltransferase (MT)‐mediated addition of a C_1_ unit from the universal methyldonor *S*‐adenosylmethionine (SAM, **4**) to the linear diphosphate terpene precursor prior to TC‐catalyzed scaffold formation (Figure 1c). The first example was the biosynthesis of homomonoterpene 2‐methylisoborneol (**5**, C_11_) which is generated via the addition of a methyl group to GPP, catalyzed by ScoMT. The modified precursor 2‐methyl GPP (**6**) is subsequently cyclized by ScoTC.^[^ ^20^, ^21^, ^22^, ^23^, ^24^, ^25^ ^]^ The pathway to **5** has also been found in different bacterial phyla including actinobacteria,^[^ ^20^ ^]^ cyanobacteria,^[^ ^22^ ^]^ and myxobacteria.^[^ ^23^ ^]^ The first pathway to C_16_ homosesquiterpenes was elucidated for sodorifen (**7**) in the proteobacterium *Serratia plymuthica*. The pathway comprises C_1_ addition to FPP by SodC, which besides methyl transfer catalyzes a complex precyclization of the substrate yielding presodorifen diphosphate (**8a**). Intermediate **8a** is subsequently further cyclized by SodD.^[^ ^26^, ^27^ ^]^ Intriguingly, homologous pathways have been identified in several proteobacteria in which SodC homologs also produce intermediate **8a**, but co‐encoded homologs of SodD catalyze distinct cyclization reactions, producing a series of structurally unusual homosesquiterpenes, collectively referred to as the “Greek philosophers homoterpenes”.^[^ ^28^, ^29^ ^]^ We have recently shown that (Figures S5A,B, S6) distinct homoterpene pathways are also widely encoded in the phylum of actinomycetota, producing simple methyl analogs of common sesquiterpenes such as kantenol (**9**). Here, C_1_ addition via a novel family of MTs (SvaMT family) generates prekantenol diphosphate (**10**), a linear double bond isomer of FPP, which is subsequently cyclized by SvaTC and homologs to a variety of germacranes and eudesmanes.^[^ ^30^ ^]^ Despite being prolific producers of diverse bioactive and pharmaceutically important terpenes,^[^ ^31^, ^32^ ^]^ no biosynthetic pathway to homoterpenes has yet been reported from the kingdom of fungi. Using a targeted genomic data mining strategy in combination with in vitro pathway reconstitution, we here report the first case for homoterpene biosynthesis in fungi.

Publicly available genomic databases were screened for fungal genomes containing biosynthetic gene clusters (BGCs) encoding for a TC and MT pair. This search revealed the cryptic *ndi* BGC in the plant‐pathogenic fungus *Neonectria ditissima* (Figure 2a, Table S1). *Ndi* is composed of a type I TC (NdiTC) and a class I MT (NdiMT), which are flanked by regulatory elements (NdiR1, NdiR2, NdiR3) and a transporter (NdiT). A BLAST search against the Uniprot database showed that NdiTC shares 31% amino acid sequence identity (aaSI) with the multiproduct‐forming sesquiterpene synthase COP4 from the basidiomycete fungus *Coprinopsis cinerea*,^[^ ^33^ ^]^ and that NdiMT shares 30% aaSI with the malonyl‐ACP O‐methyltransferase BioC, which is involved in biotin biosynthesis.^[^ ^34^ ^]^ To further assess whether the NdiMT‐NdiTC pair might constitute a bona fide fungal homoterpene biosynthetic pathway, NdiMT was compared with MTs from characterized bacterial homoterpene pathways^[^ ^28^, ^29^ ^]^ by multiple sequence alignment. Despite a low overall aaSI, with the closest match being PlMTα from *Pseudomonas lini* (WP_04 839 6718) at 42%, the alignment revealed conservation of several residues previously implicated in substrate recognition and catalysis,^[^ ^35^ ^]^ including diphosphate‐binding motifs (Figure S1).

**Figure 2:**
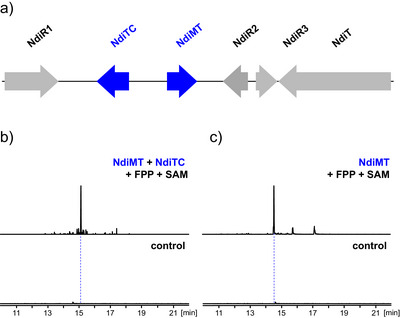
a) Depiction of *ndi* BGC. b) Extracted ion chromatogram (EIC, 218 *m*/*z*) of enzyme reaction NdiMT + NdiTC with FPP and SAM. Control (FPP + SAM without enzyme). c) EIC (236 *m*/*z*) of enzyme reaction NdiMT with FPP and SAM. Control (FPP + SAM without enzyme). Samples were treated with calf intestinal phosphatase (CIP) prior to extraction.

To determine the function of NdiMT and NdiTC, corresponding synthetic genes were purchased and expressed in *Escherichia coli* BL21. Whereas expression of NdiTC led to soluble protein in high yield and purity (Figure S2A‐C), NdiMT exhibited a low expression efficiency in several tested microbial hosts with the best result obtained for *E. coli* BL21 (Figure S2D‐F). Recombinant enzymes NdiMT and NdiTC were co‐incubated with GPP, FPP, and GGPP and subjected to gas chromatography‐mass spectrometry (GC‐MS) analysis after hexane extraction. Whereas no conversion of GPP or GGPP was detected (Figure S3), a specific product accumulated in the reaction with FPP (Figure 2b).

Evaluation of its EI mass spectrum (EI MS) suggested the presence of a homosesquiterpene, judging from a molecular ion of 218 *m*/*z* (Figure S4A). To determine the order of enzymatic steps, NdiMT and NdiTC were separately incubated with FPP. Whereas NdiTC did not convert FPP (Figure S5B), the reaction of NdiMT in the presence of SAM yielded a distinct product with 236 *m*/*z* (Figure 2c, Figure S4B). In line with these findings, NdiTC or NdiMT did not convert GPP or GGPP (Figure S2A‐C, S6). Obtained results indicate that NdiMT and NdiTC are functional and constitute the first biochemical pathway to homoterpenes in fungi.

The EI MS of the product generated by NdiMT displayed a characteristic pattern with a dominant base peak at *m*/*z* 137 (Figure S4B). A comparable spectrum has previously been reported for presodorifen alcohol (**8b**), the diphosphate hydrolyzed derivative of **8a** (Figure 1c).^[^ ^26^ ^]^ This unexpected observation suggested that NdiMT produces the same methyl‐modified diphosphate intermediate as described for the pathway to “Greek philosophers homoterpenes” in proteobacteria. To test this hypothesis, the presodorifen diphosphate synthase PchlMT^[^ ^29^ ^]^ from *Pseudomonas chlororaphis* DSM 6698 (WP_037006074.1) was amplified from genomic DNA, cloned, and heterologously expressed in *E. coli* BL21 (Figure S7). The product obtained from FPP and SAM was then compared to the NdiMT‐generated product. Both compounds exhibited identical retention times (Figure 3a) and EI MSs (Figure 3b).

**Figure 3:**
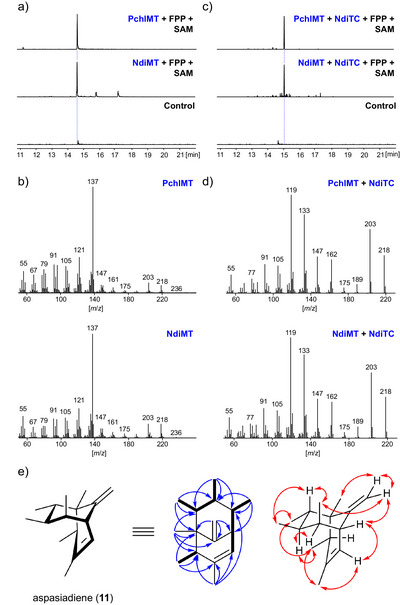
a) EICs (236 *m*/*z*) for reactions of PchlMT and NdiMT with FPP + SAM. Samples were treated with CIP prior to extraction. b) EI MS comparison of PchlMT and NdiMT generated products. c) EICs (218 *m*/*z*) for reactions of PchlMT and NdiMT with NdiTC incubated with FPP and SAM. d) EI MS comparison of PchlMT + NdiTC and NdiMT + NdiTC generated products. e) Structure of aspasiadiene (**11**) with key ^1^H,^1^H‐COSY (bold black lines), ^1^H,^13^C‐HMBC (single headed arrows), and ^1^H,^1^H‐NOESY/1D NOE (double headed arrows) correlations.

Consistent with these findings, NdiTC converted the PchlMT‐derived product into the same homosesquiterpene observed when the NdiMT product served as the substrate (Figures 3c,d), confirming that NdiMT produces presodorifen.

To elucidate the structure of the NdiTC‐generated product, preparative‐scale enzyme reactions were performed. Due to the low expression yield of NdiMT, PchlMT was used as an alternative for large‐scale production. In total, 0.5 mg of product was obtained from 16 L of PchlMT and 16 L of NdiTC expression cultures after purification by repeated column chromatography on AgNO_3_‐coated silica gel. The structure was determined by comprehensive 1D and 2D nuclear magnetic resonance (NMR) spectroscopy, including ^1^H, ^13^C, ^1^H,^1^H‐COSY, HSQC, HMBC, NOESY, and 1D NOE experiments. Based on these analyses, the structure of the NdiTC product was assigned as aspasiadiene (**11**), an unprecedented heptamethyl bicyclo[3.3.1]nonane hydrocarbon (Figure 3e, Table S2, Figure S8‐S22).

The results demonstrate that NdiMT and NdiTC constitute a functional homoterpene biosynthetic pathway in the ascomycete fungus *N. ditissima*, leading to the unique homosesquiterpene aspasiadiene (**11**), which is structurally distinct from other presodorifen diphosphate‐derived homoterpenes reported from proteobacterial pathways (Figure 4a). Given the conserved function of NdiMT but divergent function of NdiTC relative to their bacterial counterparts, their evolutionary relationships were investigated through phylogenetic analyses. NdiMT was compared with SodC^[^ ^26^, ^27^ ^]^ and homologs,^[^ ^28^, ^29^ ^]^ the 2‐GPP‐producing ScoMT,^[^ ^25^ ^]^ the prekantenol‐producing SvaMT and homologs,^[^ ^30^ ^]^ as well as BezA, a recently reported C6 specific GPP MT from benzastatin biosynthesis.^[^ ^36^ ^]^ NdiMT clustered within the same clade as bacterial SodC and its homologs, which produce presodorifen diphosphate, suggesting acquisition via horizontal gene transfer from bacteria (Figure 4b). In contrast, NdiTC appears to have evolved independently within the fungal lineage. Phylogenetic analysis compared NdiTC with canonical type I TCs from bacteria and fungi,^[^ ^37^ ^]^ TCs from proteobacterial (SodD^[^ ^26^, ^27^ ^]^ and homologs^[^ ^28^, ^29^ ^]^) and actinobacterial (SvaTC and homologs^[^ ^30^ ^]^) homoterpene pathways, as well as ScoTC^[^ ^24^ ^]^ from 2‐methylisoborneol biosynthesis. Unlike NdiMT, which grouped with SodC and its homologs, NdiTC did not cluster with SodD and related enzymes but instead formed a distinct clade with canonical fungal TCs, including aristolochene synthase^[^ ^38^ ^]^ from *Penicillium roqueforti*, daucadiene synthase^[^ ^39^ ^]^ from *Aspergillus aculeatus*, and α‐acorenol synthase^[^ ^40^ ^]^ from *Gibberella fujikuroi* (Figure 4c). This finding is further underpinned by the observation that NdiTC shares less than 16% aaSI to SodD and homologs (Figure S23). Together, these findings suggest that the discovered fungal homoterpene pathway emerged through recruitment of a bacterial derived MT and its functional integration with a TC that evolved convergently in fungi, resulting in a unique biosynthetic route. Bacterial homoterpenes produced by SodD and its homologs from presodorifen are formed through an intricate fragmentation–recombination mechanism.^[^ ^41^, ^42^ ^]^

**Figure 4:**
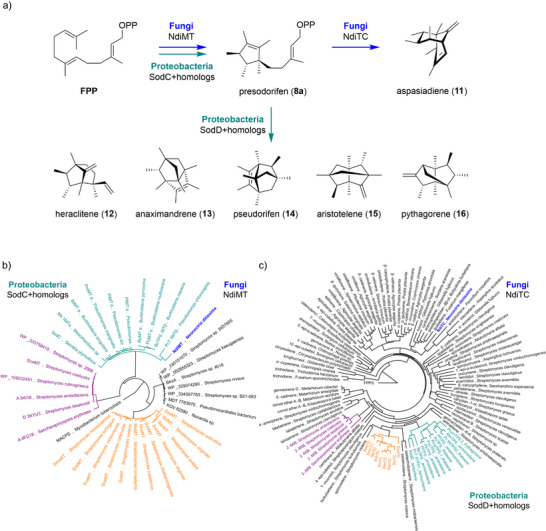
a) Comparison of proteobacterial pathways to “Greek philosophers homoterpenes” (**12**–**16**) and the discovered fungal pathway to **11** via shared intermediate presodorifen (**8a**). b) Phylogenetic tree for NdiMT (blue), SodC and homologs (cyan), C6‐GPP MT BezA (black), ScoMT family (purple), and SvaMT family (orange). The tree was rooted to mycolic acid cyclopropane synthase (MACPS, 1L1E). c) Phylogenetic tree of NdiTC (blue), SodD and homologs (cyan), ScoTC family (purple), SvaTC family (orange), and canonical type I TCs from bacteria and fungi (black). The tree was rooted to FPP synthase (FPPS, 1RQI).

Although the catalytic mechanism of NdiTC remains to be elucidated, the structure of aspasiadiene likewise suggests an elaborate cyclization chemistry (Figure S24) that, given NdiTC's evolutionary separation from SodD, likely arose through convergent evolution in the fungal kingdom.

Taken together, we report the first homoterpene biosynthetic pathway in the kingdom of fungi. Through genome mining and biochemical analysis, we elucidated the function of a MT–TC pair encoded in the genome of *N. ditissima*. NdiMT is functionally and evolutionarily related to bacterial counterparts and produces the intermediate presodorifen diphosphate, whereas NdiTC has evolved convergently within the fungus to generate a novel heptamethyl‐substituted bicyclo[3.3.1]nonene homoterpene. The results demonstrate that homoterpene biosynthesis via the addition of a C_1_ unit from SAM to terpene diphosphate precursors prior to TC‐mediated scaffold formation, extends beyond the bacterial domain to eukaryotes. Together with recent findings on the widespread occurrence of such pathways in bacteria, our results indicate that terpene methylations are more common in nature than previously recognized, potentially serving as a strategy to fine‐tune biological properties and mediate chemical communication. *N. ditissima* is a widespread fungal pathogen that causes European canker, a severe and economically important disease of apple and other woody plants.^[^ ^43^ ^]^ Although its ecological function remains to be determined, the discovered structurally unique homoterpene aspasiadiene may function as an important interspecies signal in pathogen–plant interaction and might influence host physiology or surrounding microbial communities during infection.

## Supporting Information

Supplementary Figures and Tables, experimental procedures, and compound characterization data is provided.

## Conflict of Interests

The authors declare no conflict of interest.

## Supporting information



Supporting Information

## Data Availability

The data that support the findings of this study are available from the corresponding author upon reasonable request.
