# Supplementary material for: Homoterpene Biosynthesis in Fungi
Source: Angew Chem Int Ed Engl. 2025 Nov 17;64(52):e17837. doi: 10.1002/anie.202517837 (PMC12723460; doi:10.1002/anie.202517837)
Supplement: Supplementary file 1 — Supporting Information [file ANIE-64-e17837-s001.docx]

**Supporting Information**

Homoterpene Biosynthesis in Fungi

Lin Zhou, Tatjana Reuter, Kiara Schumann, Markus Mayer, Dominik Matthias Hanauska, Lena Barra*

Table of contents

[Part I. Supporting Figures to Main Text 1](#_Toc205932375)

[Table S1. Overview of ndi BGC from *Neonectria ditissima* 1](#_Toc205932376)

[Figure S1. Multiple sequence alignment of NdiMT and selected MTs from bacterial homoterpene pathways. 1](#_Toc205932377)

[Figure S2. SDS-PAGE and intact protein mass spectrometry analysis of NdiMT and NdiTC. 2](#_Toc205932378)

[Figure S3. TICs of *in vitro* reactions with NdiMT+NdiTC 2](#_Toc205932379)

[Figure S4. EI MS of NdiMT+NdiTC and NdiMT generated products. 3](#_Toc205932380)

[Figure S5. TICs of *in vitro* reactions with NdiTC 3](#_Toc205932381)

[Figure S6. TICs of *in vitro* reactions with NdiMT 3](#_Toc205932382)

[Figure S7. SDS-PAGE analysis of purified His6-tagged PchlMT 4](#_Toc205932384)

[Figure S8. NMR data assignments and structure elucidation of aspasiadiene (11) 5](#_Toc205932385)

[Table S2. NMR data of aspasiadiene (11) 5](#_Toc205932386)

[Figure S9. ^1^H-NMR spectrum of aspasiadiene (11) 6](#_Toc205932387)

[Figure S10. ^13^C-NMR spectrum of aspasiadiene (11) 7](#_Toc205932388)

[Figure S11. ^1^H,^1^H-COSY spectrum of aspasiadiene (11) 8](#_Toc205932389)

[Figure S12. ^1^H,^13^C-HSQC spectrum of aspasiadiene (11) 9](#_Toc205932390)

[Figure S13. ^1^H,^13^C-HMBC spectrum of aspasiadiene (11) 10](#_Toc205932391)

[Figure S14. ^1^H,^1^H-NOESY spectrum of aspasiadiene (11) 11](#_Toc205932392)

[Figure S15-S22. 1D gradient NOESY spectra of aspasiadiene (11) 12](#_Toc205932393)-18

[Figure S23. Amino acid sequence identities between NdiTC, SodD, and homologs. 19](#_Toc205932404)

Figure S24. Proposed mechanism of NdiTC………………………………………………… 19

[Part II. Experimental Procedures 20](#_Toc205932405)

[General methods 20](#_Toc205932406)

[Strains and culture conditions 20](#_Toc205932407)

[Isolation of genomic DNA 20](#_Toc205932408)

[Synthetic genes for NdiMT and NdiTC 21](#_Toc205932409)

[Gene cloning 21](#_Toc205932411)

[Plasmid assembly 23](#_Toc205932412)

[Plasmid transformation 23](#_Toc205932413)

[Table S3. Overview of targeted genes and generated expression plasmids 23](#_Toc205932414)

[Table S4. Primers used in this study. 23](#_Toc205932415)

[Expression of NdiTC, NdiMT, and PchlMT 24](#_Toc205932416)

[Purification of NdiTC, NdiMT, and PchlMT 24](#_Toc205932417)

Intact protein mass spectrometry analysis of NdiTC and NdiMT………………...…………..24

[Analytical scale enzyme reactions 24](#_Toc205932418)

[Reactions of NdiMT and PchlMT 24](#_Toc205932419)

[Reaction of NdiTC 25](#_Toc205932420)

[Reactions of NdiMT+NdiTC and PchlMT+NdiTC 25](#_Toc205932421)

[Preparative scale enzyme reactions 25](#_Toc205932422)

[Reactions of PchlMT+NdiTC to isolate aspasiadiene (11) 25](#_Toc205932423)

[Isolation and purification of enzyme product 25](#_Toc205932424)

[Isolation of aspasiadiene (11) 25](#_Toc205932425)

[GC-MS analysis 25](#_Toc205932426)

[References 26](#_Toc205932427)

# Part I. Supporting Figures to Main Text

Table S1. Overview of ndi BGC from *Neonectria ditissima* including encoded genes, gene products, closest homologs in Uniprot, sequence identity to closest homolog, and proposed function.

| **Gene** | **Gene product** | **Homolog Uniprot** | **aaSI** | **Proposed function** |
| --- | --- | --- | --- | --- |
| *ndiR1* | NdiR1 | Transcription factor gsfR2 (D7PI10) | 22% | Regulatory |
| *ndiTC* | NdiTC | Linoleate 10R-lipoxygenase COP4 (A8NU13) | 31% | Type I TC |
| *ndiMT* | NdiMT | Malonyl-ACP O-methyltransferase (A3DBD7) | 30% | *C*-MT |
| *ndiR2* | NdiR2 | Aminoglycoside 3'-phosphotransferase (P0A3Y5) | 19% | Regulatory |
| *ndiR3* | NdiR3 | Zinc finger protein MSS51 (D3ZKV9) | 43% | Regulatory |
| *ndiT* | NdiT | translocator At4g32390 (Q9SUV2) | 38% | Transporter |


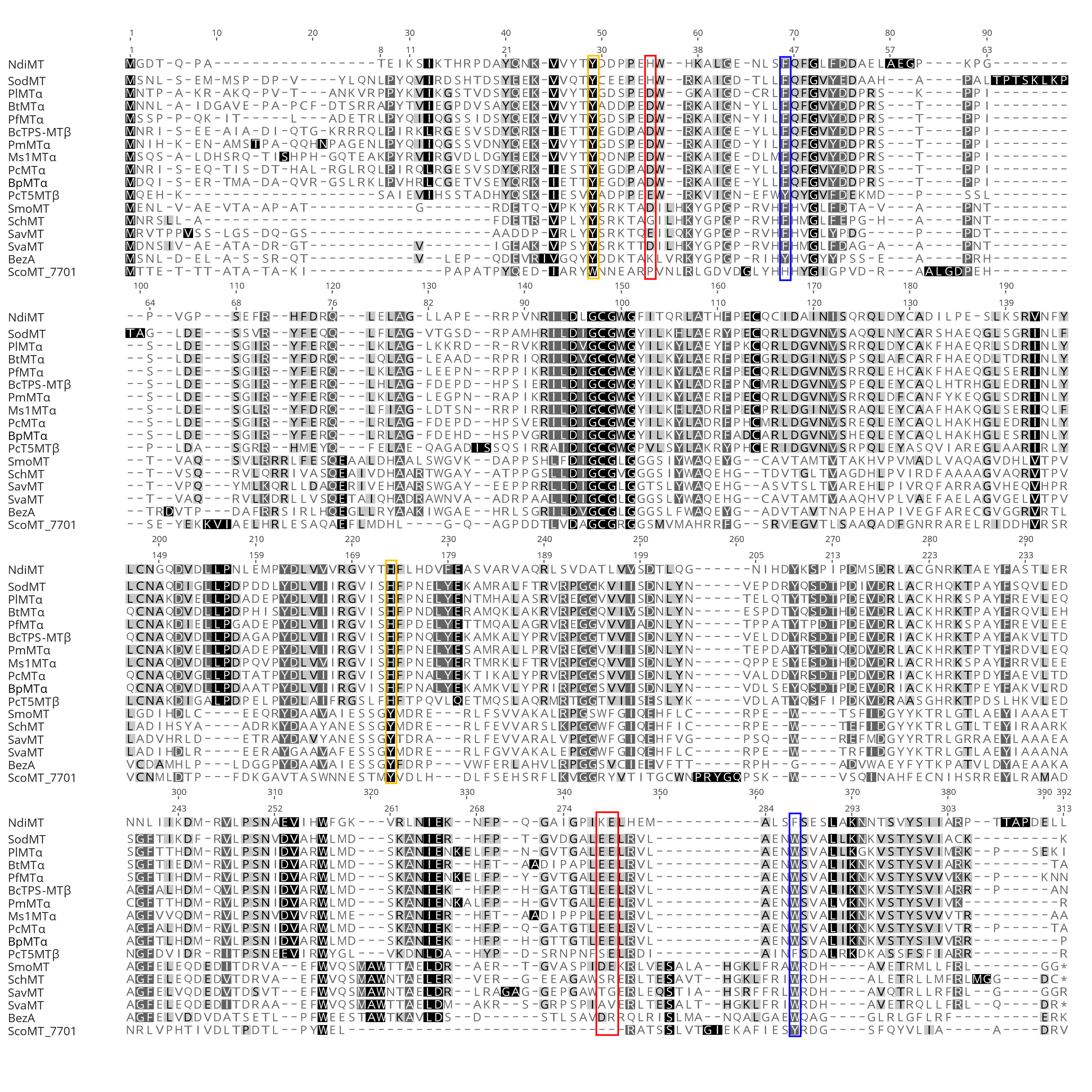


Figure S1. Multiple sequence alignment of NdiMT and selected MTs from bacterial homoterpene pathways. Boxes highlight conserved motifs proposed to be involved in substrate recognition and catalysis (red = diphosphate recognition, blue = cation stabilization, yellow = catalytic base). Accession numbers: NdiMT (KPM41701.1), SodMT (S0AH05), PlMTα (WP_048396718), BtMTα (WP_059512952), PfMTα (WP_054596444), BcTPS-MTβ (WP_059854451), PmMTα (RMT94582), Ms1MTα (WP_239369951), PcMTα (WP_012218289), BpMTα (WP_047900379), PcT5MTβ (AUG41106), SmoMT (WP_171082393), SchMT (WP_053928015), SavMT (WP_189968969), SvaMT (WP_048831493), BezA (3VC1), ScoMT_7701 (7FBH). Alignment was constructed with MUSCLE 5.1^[1]^.


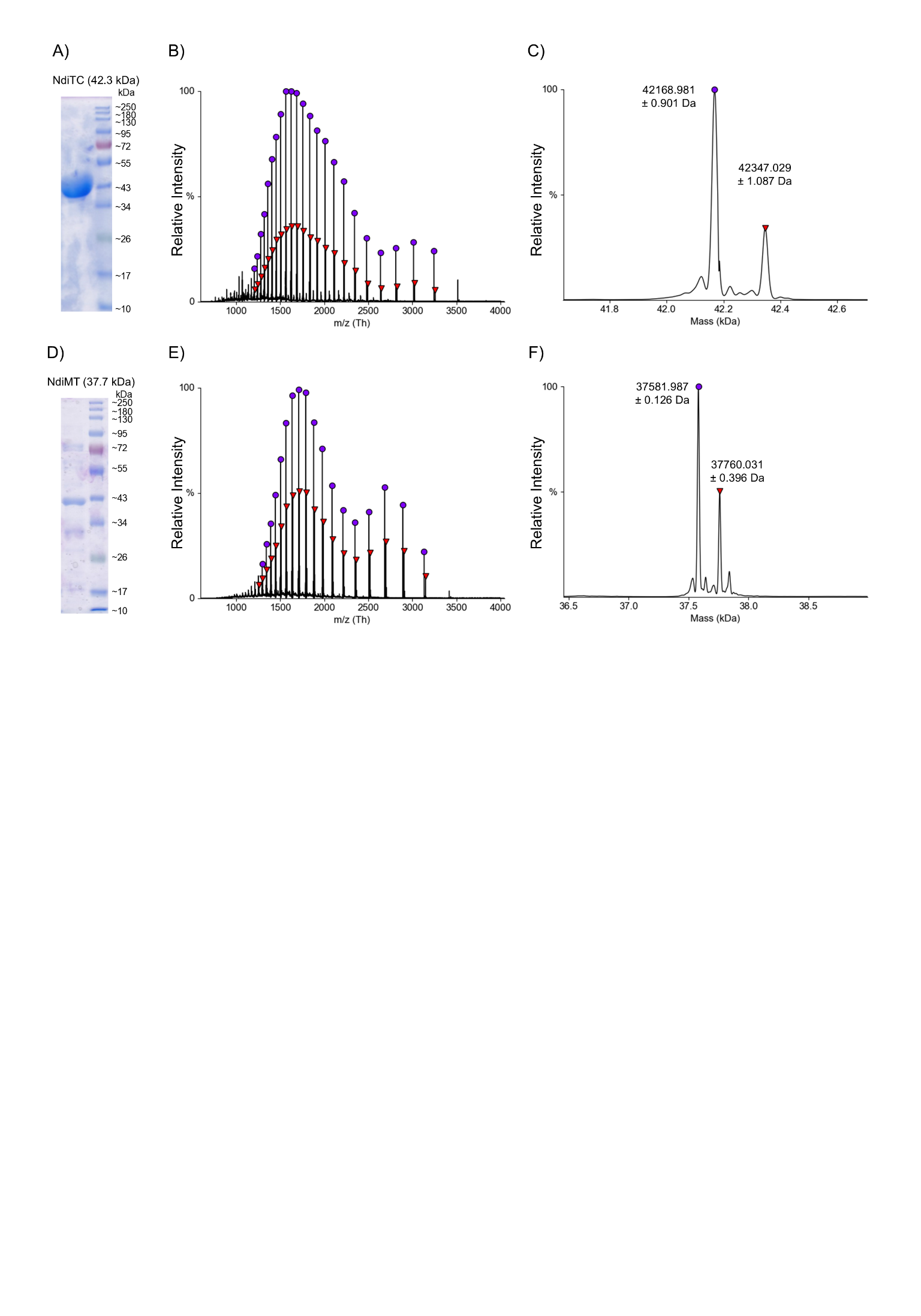


Figure S2. SDS-PAGE and intact protein mass analysis of N-terminally His6-tagged NdiTC and NdiMT expressed in *E. coli* BL21. A) SDS-PAGE analysis of NdiTC. B) Mass spectrum of NdiTC. C) Deconvoluted mass spectrum of NdiTC. D) SDS-PAGE analysis of NdiMT. E) Mass spectrum of NdiMT. F) Deconvoluted mass spectrum of NdiMT. Calculated masses after N-terminal methionine excision: NdiTC = 42.169 kDa, NdiMT = 37.582 kDa. Circles indicate demethionylated proteins; triangles indicate protein species that underwent N-terminal methionine excision and spontaneous α-*N*-6-gluconoylation^[2]^.


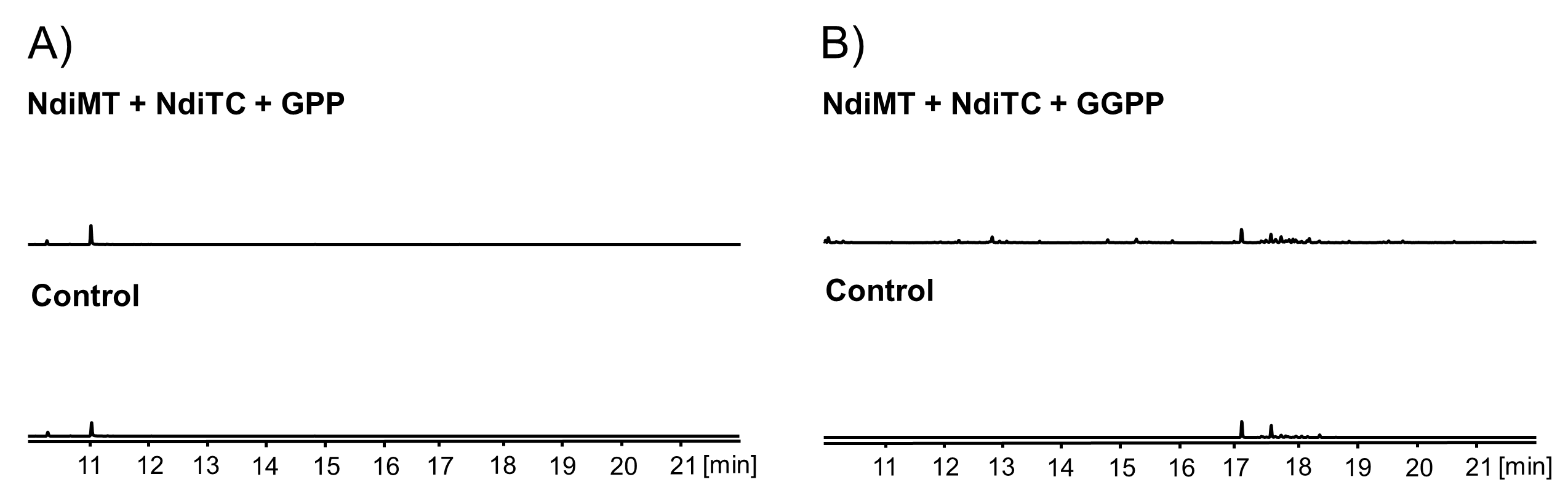


Figure S3. TICs of *in vitro* reactions with NdiMT+NdiTC**.** A) NdiMT+NdiTC+GPP, control (no enzyme). B) NdiMT+NdiTC+GGPP, control (no enzyme).


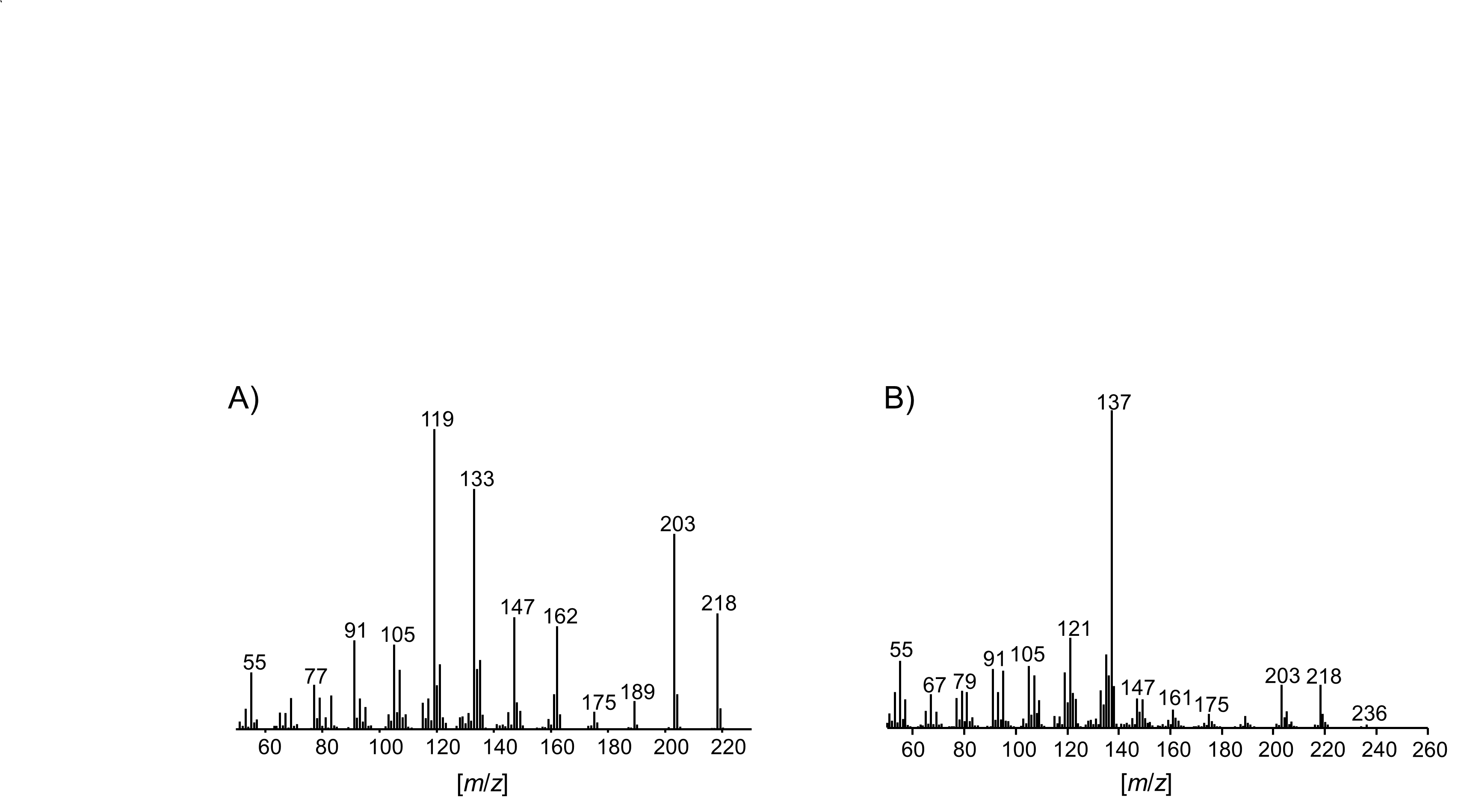


## **Figure S4.** A) EI MS of NdiMT+NdiTC product. B) EI MS of NdiMT generated product.


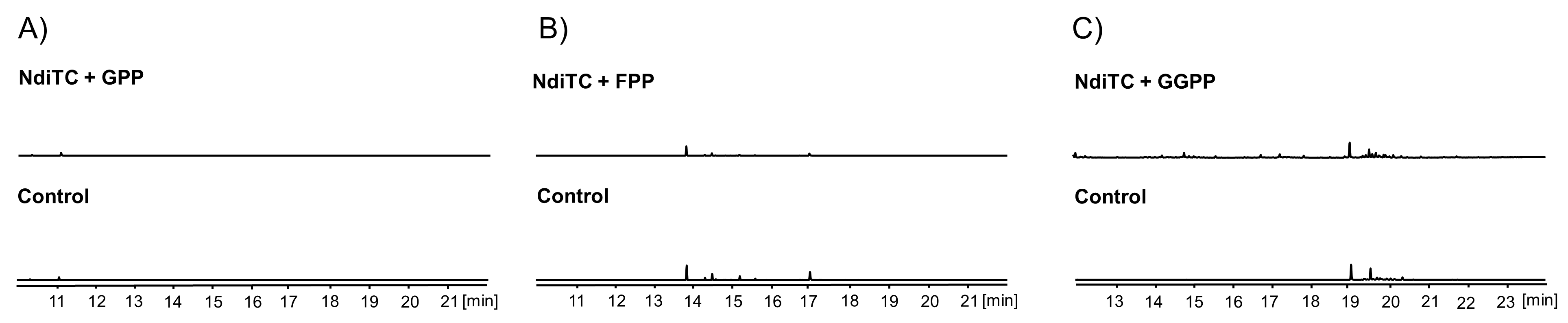


Figure S5. TICs of *in vitro* reactions with NdiTC**.** A) NdiTC+GPP, control (no enzyme). B) NdiTC+FPP, control (no enzyme). C) NdiTC+GGPP, control (no enzyme).


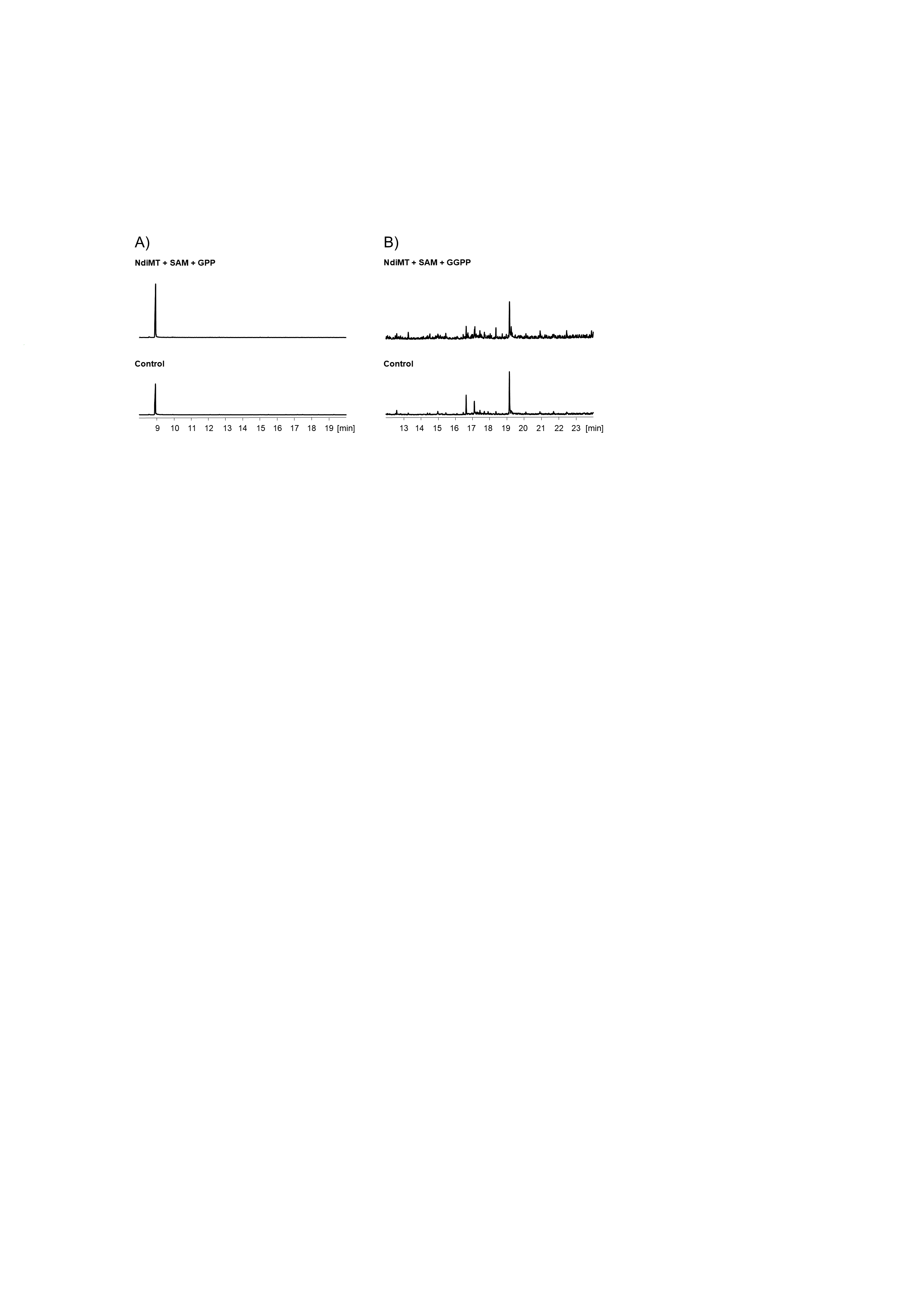


Figure S6. TICs of *in vitro* reactions with NdiMT**.** A) NdiMT+SAM+GPP, control (no enzyme). B) NdiMT+SAM+GGPP, control (no enzyme). Samples were treated with calf intestinal phosphatase (CIP) prior to extraction.


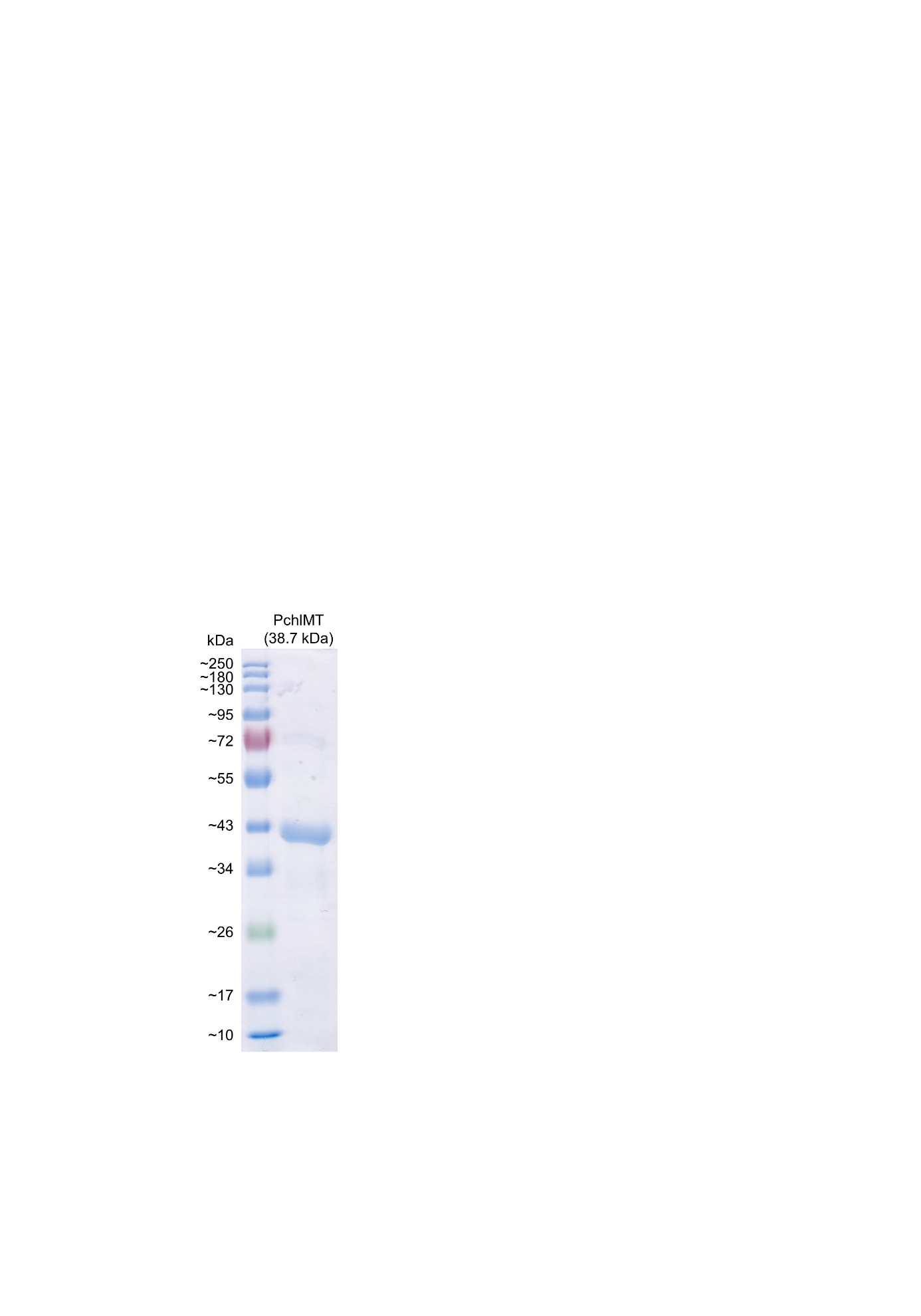


## **Figure S7**. SDS-PAGE analysis of purified N-terminally His6-tagged PchlMT expressed in *E. coli* BL21.

Figure S8. NMR data assignments and structure elucidation of aspasiadiene (11)**.** Bold lines: ^1^H,^1^H-COSY correlations. Single headed arrows: key ^1^H,^13^C-HMBC correlations. Double headed arrows: ^1^H,^1^H-NOESY and 1D NOE correlations.

Table S2. NMR data of aspasiadiene (11) in C_6_D_6_.

| C^[a]^ | type | ^13^C | ^1^H^[b]^ |
| --- | --- | --- | --- |
| 1 | CH_3_ | 16.0 | 1.01 (d, *J* = 7.8) |
| 2 | CH | 48.6 | 2.04 (q, J = 7.8) |
| 3 | Cq | 138.4 |  |
| 4 | CH | 125.6 | 5.40 (dq, *J* = 6.1, *J* = 1.2) |
| 5 | CH | 50.7 | 2.53 (dd, *J* = 6.1, *J* = 1.6) |
| 6 | CH | 45.1 | 1.23 (m) |
| 7 | CH | 32.8 | 2.14 (ddq, *J* = 12.0, *J* = 7.1, *J* = 5.0) |
| 8 | CH_3_ | 13.5 | 0.85 (d, *J* = 7.1) |
| 9 | CH | 39.9 | 1.56 (m) |
| 10 | Cq | 155.5 |  |
| 11 | Cq | 43.5 |  |
| 12 | CH_3_ | 24.2 | 1.12 (s) |
| 13 | CH_3_ | 16.1 | 0.91 (d, *J* = 7.4) |
| 14 | CH_3_ | 18.1 | 0.76 (d, *J* = 7.1) |
| 15 | CH_3_ | 21.6 | 1.59 (dd, *J* = 2.0 *J* = 1.2) |
| 16 | CH_2_ | 102.7 | 4.78 (d, *J* = 1.8) |
|  |  |  | 4.70 (d, *J* = 1.8) |

[a] Carbon numbering as shown in Figure S8. [b] Chemical shifts δ in ppm, multiplicity: s = singlet, d = doublet, m = multiplet. Coupling constants *J* are given in Hertz.

Figure S9. ^1^H-NMR spectrum of aspasiadiene (11) (600 MHz, C_6_D_6_).

Figure S10. ^13^C-NMR spectrum of aspasiadiene (11) (150 MHz, C_6_D_6_).

Figure S11. ^1^H,^1^H-COSY spectrum of aspasiadiene (11) (C_6_D_6_).

Figure S12. ^1^H,^13^C-HSQC spectrum of aspasiadiene (11) (C_6_D_6_).

Figure S13. ^1^H,^13^C-HMBC spectrum of aspasiadiene (11) (C_6_D_6_).

Figure S14. ^1^H,^1^H-NOESY spectrum of aspasiadiene (11) (C_6_D_6_).


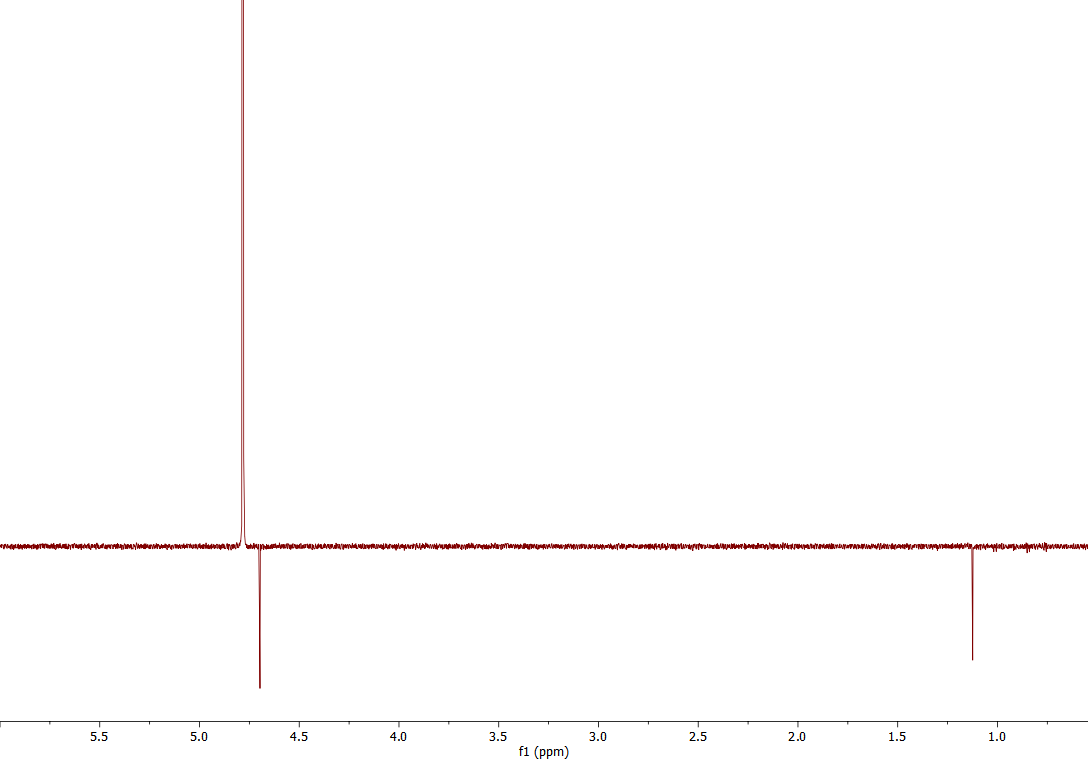


Figure S15. 1D gradient NOESY spectrum of aspasiadiene (11) (C_6_D_6_). Frequency = 4.78 ppm

**
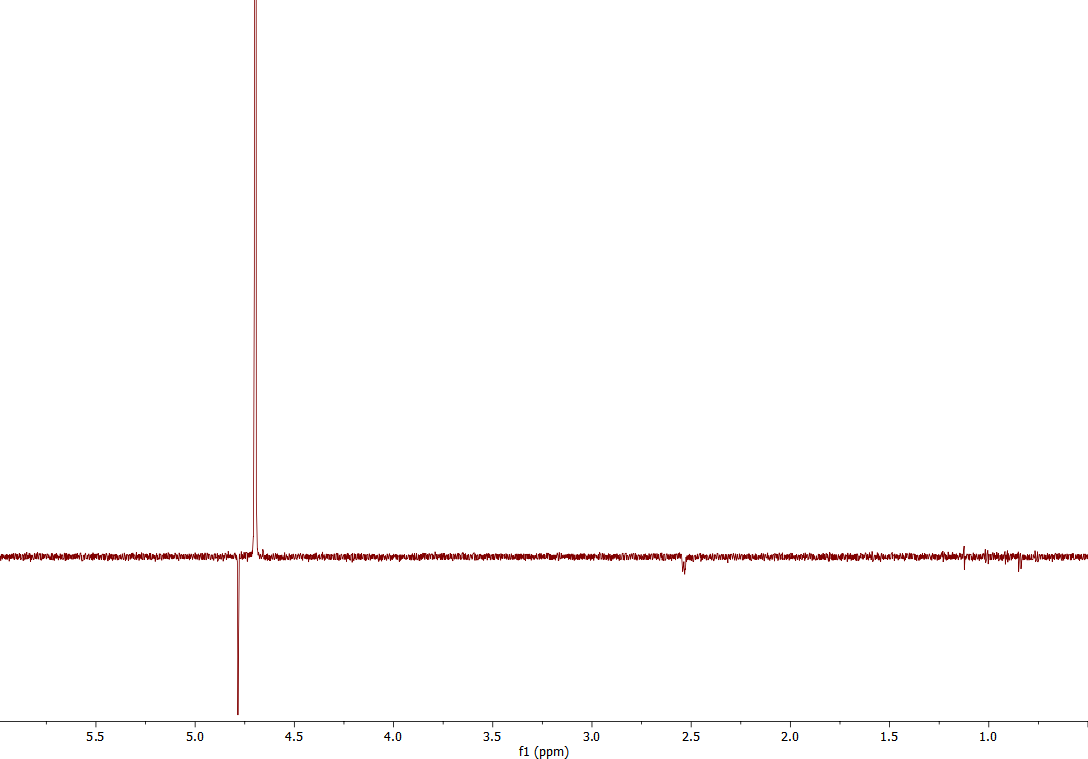
**

Figure S16. 1D gradient NOESY spectrum of aspasiadiene (11) (C_6_D_6_). Frequency = 4.70 ppm

**
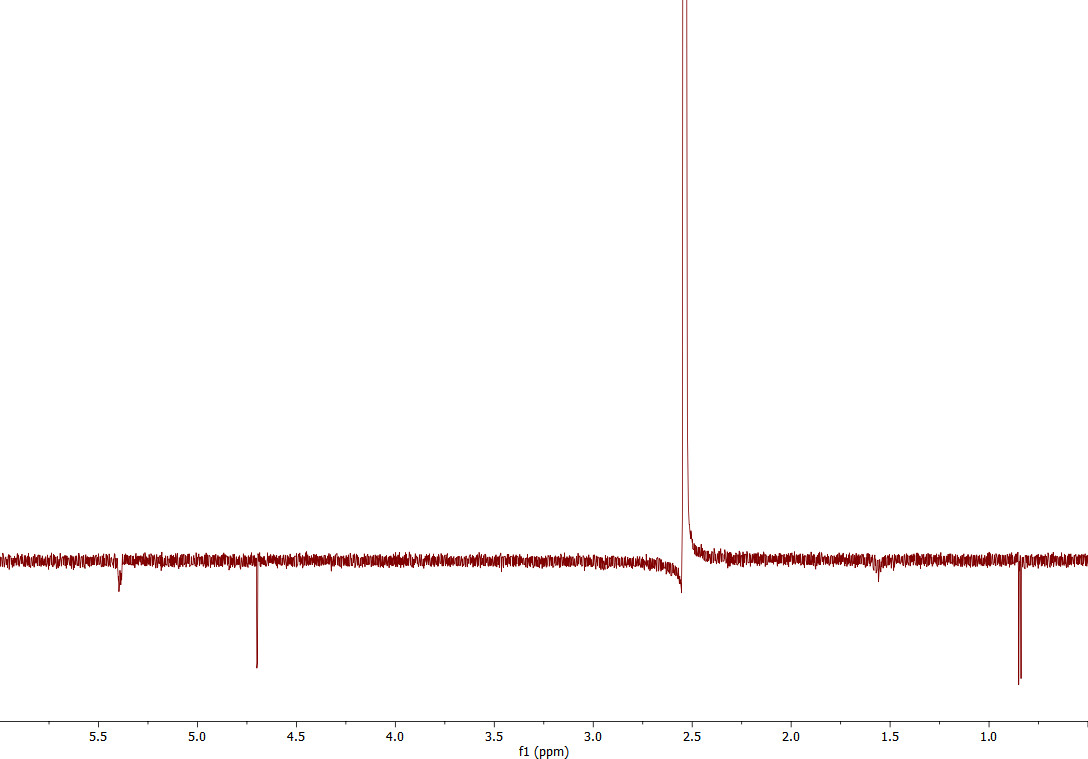
**

Figure S17. 1D gradient NOESY spectrum of aspasiadiene (11) (C_6_D_6_). Frequency = 2.53 ppm


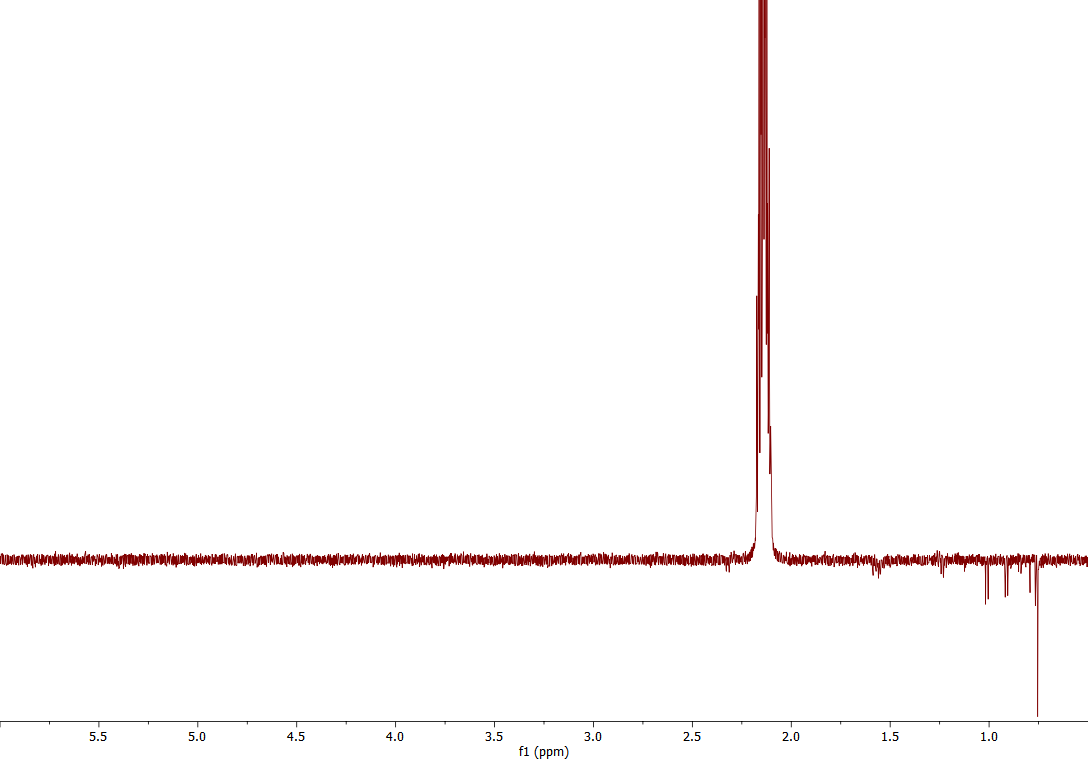


Figure S18. 1D gradient NOESY spectrum of aspasiadiene (11) (C_6_D_6_). Frequency = 2.14 ppm


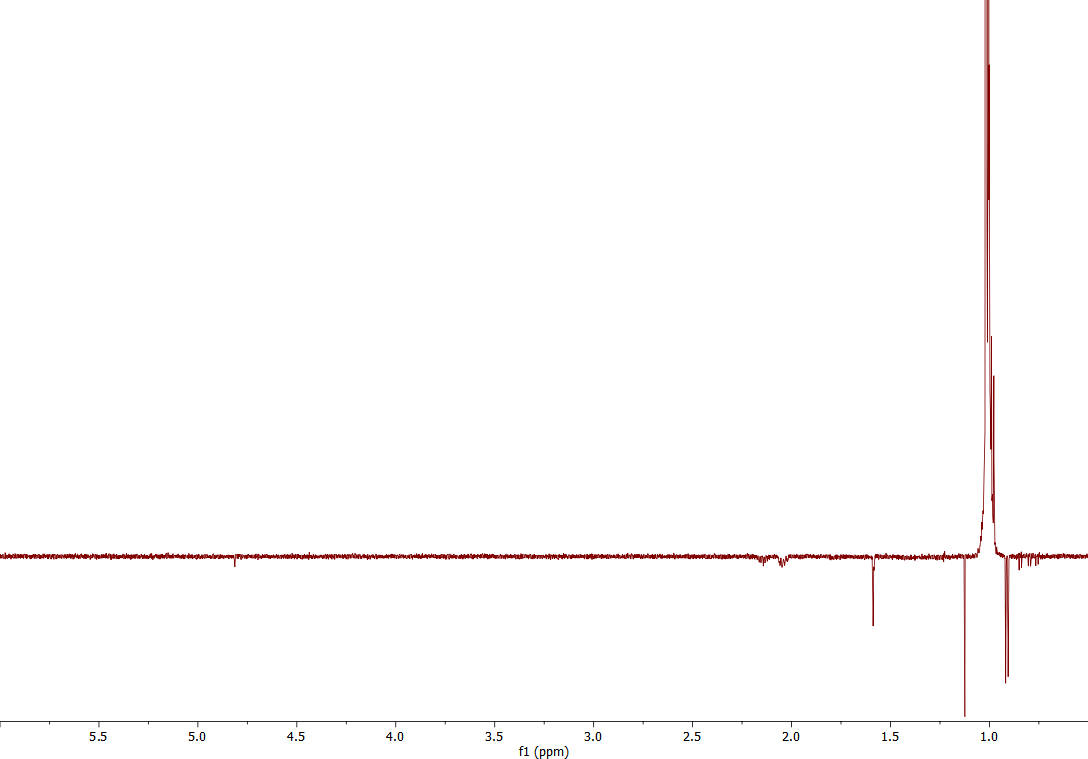


Figure S19. 1D gradient NOESY spectrum of aspasiadiene (11) (C_6_D_6_). Frequency = 1.01 ppm


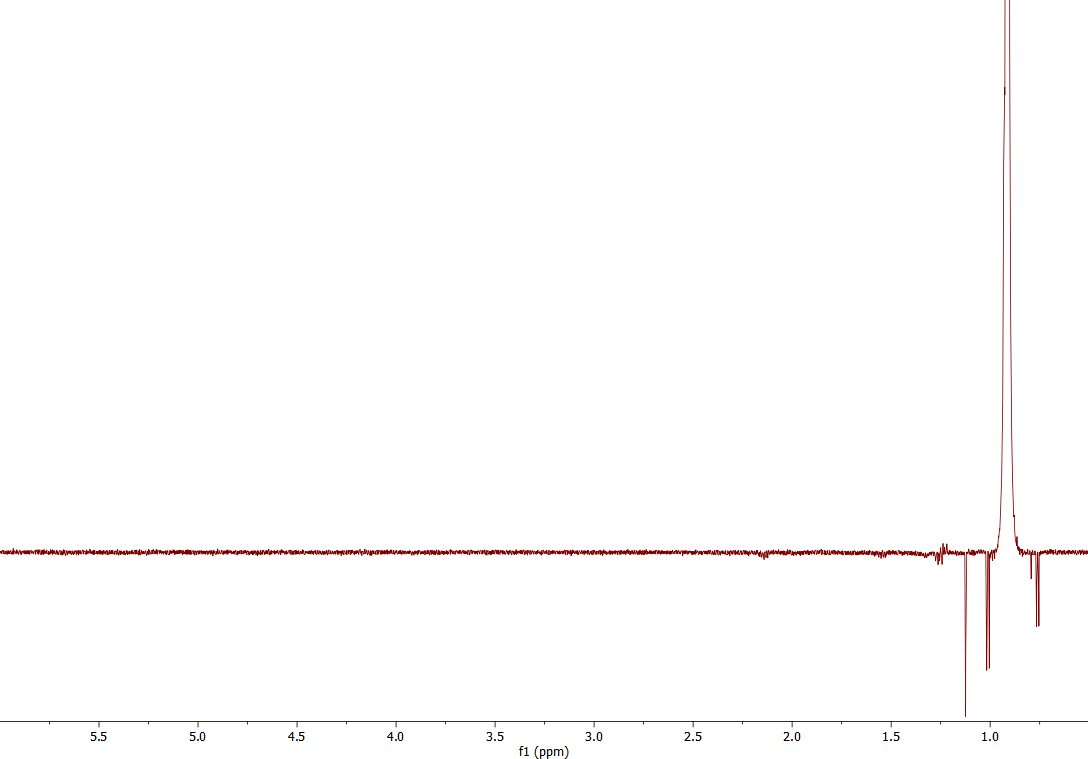


Figure S20. 1D gradient NOESY spectrum of aspasiadiene (11) (C_6_D_6_). Frequency = 0.91 ppm


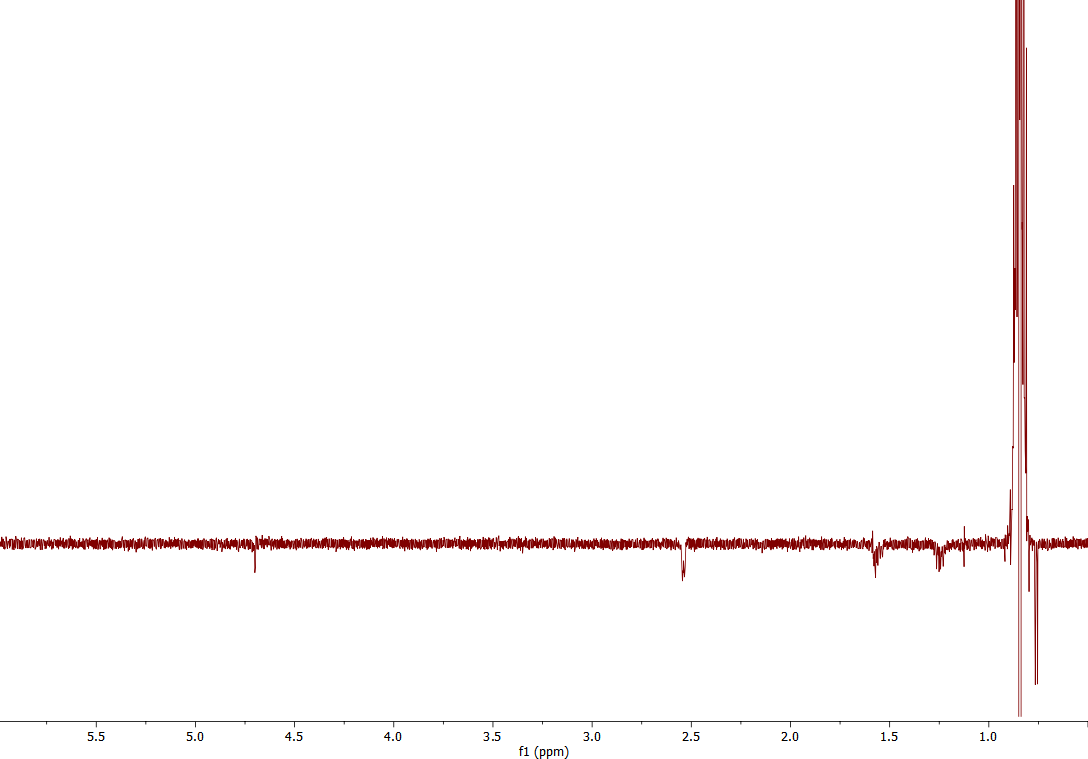


Figure S21. 1D gradient NOESY spectrum of aspasiadiene (11) (C_6_D_6_). Frequency = 0.85 ppm


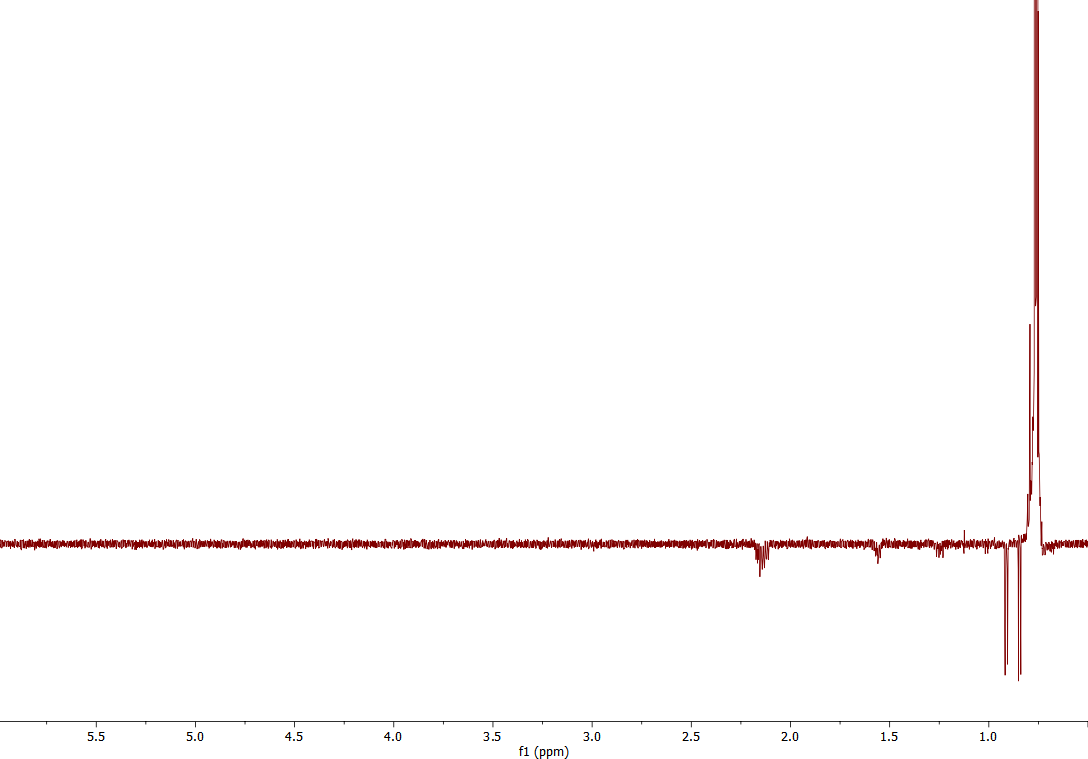


Figure S22. 1D gradient NOESY spectrum of aspasiadiene (11) (C_6_D_6_). Frequency = 0.76 ppm


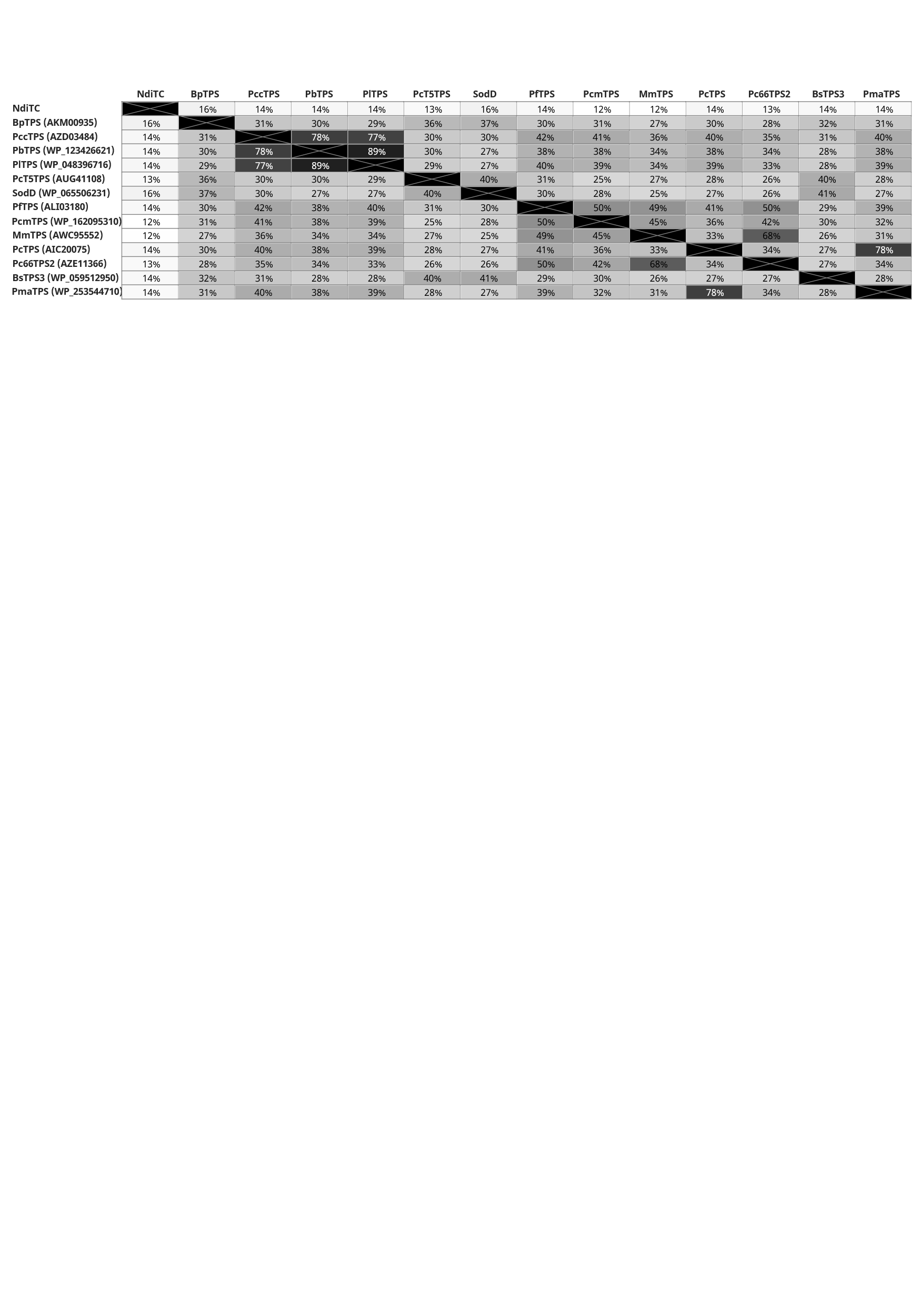


Figure S23. Amino acid sequence identities between NdiTC, SodD, and homologs from bacterial homoterpene pathways. Constructed with MUSCLE 5.1^[1]^.

Figure S24. Mechanistic proposal for the formation of aspasiadiene (11) from presodorifen (8a) by a fragmentation-recombination pathway via intermediate E. Intermediate E has been validated as a key intermediate in cyclization reactions towards sodorifen (7), anaximandrene (13), aristotelene (15), and pythagorene (16) (see reference 22 in main text).

# Part II. Experimental Procedures

## General methods

Strain *Pseudomonas chlororaphis* DSM 6698 was received from DSMZ. LB, LB-agar, media ingredients, antibiotics, isopropyl-β-D-thiogalactoside (IPTG), and ROTI^®^Garose-His/Ni NTA-Beads were purchased from Carl Roth. Chemicals including geraniol, farnesol, *S*-adenosyl-l-methionine (SAM), were purchased from Merck, TCI, or BLDpharm and used without further purification. *E. coli* strain DH5α was used for DNA propagation. Phusion High-Fidelity PCR Master Mix with GC Buffer, NdeI, XhoI, and NEBuilder HiFi DNA Assembly Master Mix were purchased from New England Biolabs. GoTaq G2 Green Master Mix, Wizard SV Gel and PCR Clean-Up System as well as Wizard Plus SV Minipreps DNA Purification System were purchased from Promega. In-Fusion Snap Assembly Master Mix was purchased from Takara. Vector pET28a-His-TEV was gifted from Prof. Dr. Jörg Hartig. Mastercyclers X50a and Nexus X2 from Eppendorf were used for all PCR reactions. All primers as well as PD-10 desalting columns were purchased from Merck. ddH_2_O was sourced from a MiliQ Synthesis A10 water purification unit from Millipore and used for buffer preparation. Silica gel 60 F_254_ plates (Sigma-Aldrich) were used for TLC. The staining solution for TLC was molybdophosphoric acid in EtOH (100 mg ml^-1^). Flash chromatography was performed with silica gel 60 M (0.04 – 0.063 mm, Macherey-Nagel). Optical rotations were recorded on a Jasco P-2000 digital polarimeter. The optical rotation parameters were (i) temperature: 25 °C, (ii) wavelength: 589 nm (sodium *D* line), and (iii) path-length: 10 cm. NMR spectra were recorded on a 600 MHz cryospectrometer (Avance NEO, Bruker). Chemical shifts were referenced to the residual proton signal of the solvent for ^1^H-NMR and the respective ^13^C signal for ^13^C-NMR. Data collections were done using Bruker TopSpin 4.2 pL0 software. Data analyses were performed with Mestrenova 15. Substrates GPP, FPP, and GGPP were chemically synthesized according to literature procedure.^[3]^

## Strains and culture conditions

*P. chlororaphis* DSM 6698 was cultivated in DSM medium 1 (5 g peptone, 3 g meat extract, 1 l distilled water, pH 7.0) at 28°C. *E. coli* DH5α and BL21(DE3) were grown in LB medium (Luria/Miller) or on LB-agar plates (Luria/Miller) at 37°C. Kanamycin was used at a concentration of 50 µg ml^-1^.

## Isolation of genomic DNA

Genomic DNA (gDNA) was isolated following standard phenol-chloroform-isoamyl alcohol (25:24:1, pH 7.5 – 8) extraction. Cells were resuspended in buffer (75 mM NaCl, 25 mM EDTA, 20 mM Tris-HCl, pH 8) and treated with lysozyme (1 mg ml^-1^) for 2 h at 37°C, followed by proteinase K (0.4 mg ml^-1^) treatment at 55°C (1 h) and addition of SDS (1%). An equal volume of phenol-chloroform-isoamyl alcohol was added and the contents were mixed by shaking. After centrifugation, the aqueous phase was transferred to a fresh tube and washed with chloroform to remove any remnant phenol. To precipitate DNA, ice-cold 100% ethanol was added to the aqueous phase and the mixture was centrifuged. The pellet was washed two times with ice-cold 70% ethanol and air-dried before resuspension in TE buffer.

## Synthetic genes for NdiMT and NdiTC

## *E. coli* codon-optimized synthetic genes of NdiMT (KPM41701) and NdiTC (KPM41696) containing overhangs allowing direct assembly with linearized pET28a-His-TEV plasmid were purchased from TWIST Bioscience and subsequently integrated into the expression vector as described below using NEBuilder HiFi DNA Assembly Master Mix.

Nucleotide sequences of codon-optimized synthetic genes (overhangs are underlined):

> *ndiMT*

AATCTTTATTTTCAGGGCCATATGGGTGACACGCAACCAGCCACAGAAATTAAATCCATCAAGACTCACCGGCCCGATGCCTATCAAAACAAAGTGGTCTACACGTACGATGATCCACCAGAACATTGGCACAAGGCACTTGGTGAAAACCTGAGCTTCCAGTTCGGTTTGTTTGATGATGCGGAATTGGCCGAAGGTCCGAAACCAGGCCCGGTAGGACCATCGGAATTCAGACACTTTGATCGACAGTTGGAACTGGCCGGCCTGCTGGCTCCGGAACGTCGTCCGGTCAATCGGATACTTGATCTCGGTTGCGGTTGGGGATTTATCACGCAGCGTCTGGCGACACACTTTCCCGAATGCCAGTGTATTGACGCCATAAATATTAGTCAGAGACAGCTTGATTACTGTGCGGATATCCTCCCCGAAAGCTTGAAGTCACGGGTTAACTTTTATTTATGCAATGGACAAGATGTGGATCTGCTGCCCAACTTAGAAATGCCATATGATCTGGTTGTCGTGAGAGGAGTGTATACCCATTTTCTGCATGATGTCTTTGAGGCATCAGTAGCTCGTGTTGCCCAGCGTCTGTCCGTGGATGCCACACTTGTGGTATCCGATACCCTGCAGGGCAACATCCATGACTATAAGTCGCCGATTCCTGACATGTCAGATAGACTGGCTTGTGGCAATCGAAAAACGGCCGAGTATTTCGCCAGCACACTGGAACGCAATAATCTGATTATAAAAGATATGCGCGTCCTCCCATCAAATGCAGAGGTGATTCATTGGTTTGGAAAAGTACGCCTGAATATTGAAAAGAACTTCCCTCAGGGTGCTATAGGTCCCATTAAAGAGCTGCATGAAATGGCGCTGAGTTTTAGTGAAAGTTTGGCAAAAAACAATACCTCGGTTTATTCAATTATTGCACGCCCCACAACAGCCCCCGATGAACTTCTGTGACTCGAGCACCACCACCACCACC

> *ndiTC*

AATCTTTATTTTCAGGGCCATATGACCGGCAATGAAAGTTTCCTGCCGCCAGGTACGGTATTCAACCTTCCGGAAGTTATTACTCGTCTGCCGGAGGCATGCCATGGCGACTGTGCGCAACTCGACCATACCAGTGATGCGTTAGCACGACAGTATCTTGTTGATTATTTTGGCGATGAGGAACGTGCAGATCGATACCTGCGCCAACGAATCTCGCACTTTGCGTGTATCTGCTATCCGTATTCCTCAAGCGAACGGGCGCAATCACTGACTAACTTAATGATTCCGGTAACGATTTTCGACGACACCTTTAGCCGTCCGGATACGCAAGAAGATCTCGCGGCTGCTACAGCGCTTCATGACGATTGGTGCGCTGTTTTCAAGGGTCACCGCCCGCCACCGCATGGCAATAGTGCTTTCCTGCTCGTTTATAATGCTATCGAAGCTGCGGCTGCTCGTATGTCTGAACATCTGGCTGCCCGCGTGCGTCAAGCATGGTCGGACATGGCAGCAAGCCATCTCGATGAGGCAGTGCTGCGGAACAATAACATTGCCCTGTCGTATGAGCAGTACATGGAAAAGCGTTTAACTAACGTGTACGGCTGGTGGCTGACGACCCATGTAGAATATGCCAAGGGCATTGATCTGGGTGAGTTATCTAATCACCCTGAGATAATGGCTGTGCGCCGGGCGGCGATTAACCATGTGACTTTAGTAAATGATTTATATAGCTTTCCCAAAGAGTTAGACGCGAAAGAGGCGATGATGAATGCCATCCTGGTTCTTATGCGTCGTGAAAATTTGAATCTGCAGCAGAGCATCGATAAAGTGGTTGACAACATACACCAAGCAGAGCTGGATTTTATTAGTGCTCGCGATAACATCCTTCAAGGGCAGCAGGGACAGTTGTCGGATATAGGGGCCTATCTGGATGGTGTAGGCTACGTCATGACTGGCAATCTGCGCTGGAGCCAAATGTCAACCCGTTACTTCGGAGACGATCATGATGGGGCCCGCATTACTAGTGGTCCGATTACGATTACCCCGAAACCAACAGTTCATGCACCAGCTCCGAGAGCCGAGGTGTAACTCGAGCACCACCACCACCACC

## Gene cloning

The nucleodite sequence encoding for PchlMT (WP_037006074) was obtained by polymerase chain reaction (PCR) using Phusion High-Fidelity PCR Master Mix with GC Buffer (New England Biolabs) supplemented with 10% DMSO and isolated gDNA as template. Primer pairs are listed in Table S4. PCR 3-step conditions were used (initial denaturation at 98°C for 1 min, 35 cycles with denaturation at 98°C for 10 s, Annealing at 55°C for 15 s, elongation at 72°C for 45 s, final elongation step at 72°C for 5 min). PCR product was stained using Midori Green Direct Stain (NIPPON Genetics) and separated by gel electrophoresis. Product with expected size was excised and purified using Wizard SV Gel and PCR Clean-Up System (Promega).

## Plasmid assembly

Expression vector pET28a was used containing a TEV protease in place of thrombin cleavage site (pET28a-His-TEV). Targeted (synthetic) genes were integrated into NdeI/XhoI linearized pET28a-His-TEV plasmid (Table S3). Linearized vector was purified using the Wizard SV Gel and PCR Clean-Up System (Promega) after separation by agarose gel electrophoresis. Targeted genes and linearized vector were assembled using In-Fusion Snap Assembly Master Mix (Takara) or NEBuilder HiFi DNA Assembly Master Mix (New England Biolabs) and immediately transformed into ultracompetent *E. coli* DH5α by heat-shock. Transformed *E. coli* cultures were plated on LB-agar plates containing kanamycin (50 µg ml^-1^) and grown overnight at 37°C. Colonies were collected from plates and examined for successful DNA assembly by colony PCR using GoTaq G2 Green Master Mix (Promega), primer pair T7 promoter/terminator (Table S4), and a 3-step PCR program (initial denaturation at 95°C for 2 min, 35 cycles with denaturation at 95°C for 15 s, annealing at 50°C for 15 s, elongation at 72°C for 1:30 min, final elongation step at 72°C for 5min). Single colonies were selected to inoculate 10 ml LB medium with kanamycin and resulting cultures were grown overnight at 37°C with shaking. Plasmid DNA was isolated using Wizard Plus SV Minipreps DNA Purification System (Promega) and verified by sequencing.

## Plasmid transformation

Expression plasmids encoding for NdiTC, NdiMT, and PchlMT (Table S3) were transformed into ultracompetent *E. coli* BL21(DE3). The transformed cultures were selected in 5 ml LB medium containing 50 µg ml^-1^ kanamycin overnight at 37°C with shaking.

Table S3. Overview of targeted genes and generated expression plasmids in this study.

| **Organism** | **MT**  **(Acc. number)** | **MT**  **Expression Plasmid** | **TC**  **(Acc. number)** | **TC**  **Expression Plasmid** |
| --- | --- | --- | --- | --- |
| *Neonectria ditissima*  *R09 05* | NdiMT  (KPM41701) | pET28a-His-TEV-NdiMT | NdiTC  (KPM41696) | pET28a-His-TEV-NdiTC |
| *Pseudomonas chlororaphis DSM 6698* | PchlMT  (WP_037006074) | pET28a-His-TEV-PchlMT | - | - |

Table S4. Primers used in this study. Overlaps used for DNA assembly with linearized pET28a-His-TEV are underlined.

| **Primer name** | **Primer DNA sequence (5’🡪 3’)** |
| --- | --- |
| PchlMT_F  PchlMT_R  T7 promoter | TCTTTATTTTCAGGGCCATATGAGCATTCAAGCCAGAGAG  GTGGTGGTGGTGGTGCTCGAGTCATCCTGGAAATGGCTTT  TAATACGACTCACTATAGGG |
| T7 terminator | GCTAGTTATTGCTCAGCGG |

## Expression of NdiTC, NdiMT, and PchlMT

*E. coli* BL21(DE3) transformants harboring expression plasmids (Table S3) were grown in LB medium containing kanamycin (50 µg ml^-1^) at 37°C with shaking overnight. 2.5 ml of precultures were used to inoculate 1 l LB medium (50 µg ml^-1^ kanamycin). Expression cultures were incubated at 37°C with shaking until an absorbance (600 nm wavelength) of 0.7 - 0.8 was attained. Subsequently, cultures were cooled to 18°C and protein expression was induced by addition of IPTG (400 mM in ddH_2_O, 1 ml l^-1^), followed by incubation overnight at 18°C with shaking.

## Purification of NdiTC, NdiMT, and PchlMT

Expression cultures were centrifuged at 4347 g at 4°C, the supernatant was discarded, the cell pellet was washed with ddH_2_O, and resuspended in lysis buffer (50 mM Tris, 200 mM NaCl, 10 mM MgCl_2_, 10 mM imidazole, pH 7.4). Cells were lysed by sonication (Sonoplus HD4100 Bandelin, TS106, 50% Amplitude, 5 rounds of 45s on / 30s off). Cell debris was removed by centrifugation (16000 g, 30 min, 4°C) and the clarified lysate was filtered (Filtropur S 0.2) before loading onto a ROTI^®^Garose-His/Ni NTA-Beads containing plastic column that had been equilibrated with lysis buffer. The loaded protein was washed with lysis buffer (20 - 30 ml) and eluted using elution buffer (50 mM Tris, 200 mM NaCl, 10 mM MgCl_2_, 500 mM imidazole, pH 7.4). Proteins were subsequently desalted using PD-10 desalting columns (Cytiva) equilibrated with incubation buffer (50 mM Tris, 200 mM NaCl, 10 mM MgCl_2_, 10 % glycerol, pH 7.4) following the manufacturer’s instructions. Concentrations of purified proteins were determined using Nanodrop Lite Plus spectrophotometer (Thermo Scientific) and analyzed by SDS-PAGE (Figure S2, Figure S7). All protein purification steps were performed at 4°C.

**Intact protein mass spectrometry analysis of NdiTC and NdiMT**

Purified proteins were prepared as described above. Prior to mass spectrometric analysis, all samples were desalted by UHPLC on a Dionex UltiMate3000 (Thermo Fisher Scientific, Germany) using an analytical Aeris WIDEPORE C4 column (150 mm x 2.1 mm, 3.6 µm silica particle size; Phenomenex, Germany) as a stationary phase. Samples were acidified with 10% TFA to pH below 4.0. Gradient elution was performed at a flow rate of 250 µl/min using eluent A (0.02% TFA in water) and eluent B (0.02% TFA in acetonitrile/water, 80:20 v/v) with the following gradient profile: 2 min at 0% B, ramped to 40% B over 6min, then to 70% B over next 22 min, and finally to 100% B within 5 min, followed by a 5 min wash at 100% B. UV absorbance was monitored at 220 nm. The samples were analysed by direct infusion on a QE UHMR mass spectrometer (Thermo Scientific) with a flow rate of 5 µl/min. Mass spectrometric data were evaluated and deconvoluted using the UniDec Launcher Version 7.0.2 software.

## Analytical scale enzyme reactions

### Reactions of NdiMT and PchlMT

Freshly purified enzymes (10 µM) were incubated with SAM (1 mM) and the respective terpene substrate GPP, FPP, or GGPP (1 mM) in a reaction volume of 300 µl (50 mM Tris, 200 mM NaCl, 10 mM MgCl_2_, 10 % glycerol, pH 7.4) at 30°C for 12 h without agitation. Calf intestinal alkaline phosphatase (0.5 µl of 5000 U l^-1^ CIP, NEB) was added, followed by incubation at 37°C for 2 h. The reaction mixtures were extracted with 150 µl hexane. The organic layers were dried with MgSO_4_ and directly analyzed by GC-MS.

### Reaction of NdiTC

Freshly purified enzyme (10 µM) was incubated with the respective terpene substrate GPP, FPP, or GGPP (1 mM) in a reaction volume of 300 µl (50 mM Tris, 200 mM NaCl, 10 mM MgCl_2_, 10 % glycerol, pH 7.4) at 30°C for 12 h without agitation. The reaction mixtures were extracted with 200 µl hexane. The organic layers were dried with MgSO_4_ and analyzed by GC-MS.

### Reactions of NdiMT+NdiTC and PchlMT+NdiTC

Freshly purified enzymes (10 µM each) were incubated with FPP (1 mM) and SAM (1 mM) in a reaction volume of 300 µl (50 mM Tris, 200 mM NaCl, 10 mM MgCl_2_, 10 % glycerol, pH 7.4) at 30°C for 12 h without agitation. The reaction mixtures were extracted with 200 µl hexane. The organic layers were dried with MgSO_4_ and analyzed by GC-MS.

## Preparative scale enzyme reactions

### Reactions of PchlMT+NdiTC to isolate aspasiadiene (11)

Freshly purified PchlMT (15 µM) and NdiTC (15 µM) from 16 l expression culture each were incubated with SAM (1.2 mM, 81.3 mg) and FPP (1 mM, 54.2 mg) in batches of 1.5 ml reactions in buffer (50 mM Tris, 200 mM NaCl, 10 mM MgCl_2_, 10 % glycerol, pH 7.4) at 20°C for 12 – 16 h without agitation. Reaction mixtures were extracted with pentane, organic layers combined, dried over MgSO_4_, and concentrated under reduced pressure. The crude extract was subjected to further purification as outlined below.

## Isolation and purification of enzyme product

### Isolation of aspasiadiene (11)

The crude extract was purified by repeated column chromatography (5% AgNO_3_-coated silica gel) using pentane/diethylether (15:1). Aspasiadiene (**11**) was isolated as a colorless oil (0.5 mg).

TLC (pentane): *R*_f_ = 0.90. GC (OPTIMA 5 MS): *I* = 1558. Optical rotation: [α]_D_^25^ = +32.5 (c = 0.002 M, C_6_D_6_). NMR data are given in Table S2.

## GC-MS analysis

GC-MS analyses were carried out on a TRACE GC Ultra/ISQ gas chromatography/mass detector (Thermo Scientific). The GC was equipped with a OPTIMA 5 MS column (30 m, 0.25 mm inner diameter, 0.25 µm film; Macherey-Nagel) and operated using the following settings: (1) inlet pressure, 70 kPa, H_2_ gas at 50 ml min^-1^; (2) injection volume, 1 µl; (3) temperature program, 2 min at 50°C then increasing 10°C min^-1^ to 280°C, hold 3 min; (4) splitless; and (5) carrier gas H_2_ at 0.7 ml min^-1^. The MS was operated with the following settings: (1) source, 220°C; (2) transfer line, 290°C; (3) electron energy, 70 eV; and (4) solvent delay, 7.5 min. Data collection and analyses were performed using XCalibur QualBrowser software. To determine retention indexes, GC-MS analyses were carried out on a HP6890 gas chromatography/ 5973 Mass Selective Detector (HEWLETT PACKARD). The GC was equipped with a OPTIMA 5 MS column (30 m, 0.25 mm inner diameter, 0.25 µm film; Macherey-Nagel) and operated using the following settings: (1) inlet pressure, 0.123 bar, H_2_ gas at 58 ml min^-1^; (2) injection volume, 1 µl; (3) temperature program, 2 min at 50°C then increasing 10°C min^-1^ to 280°C, hold 2 min; (4) splitless; and (5) carrier gas H_2_ at 1.2 ml min^-1^. The MS was operated with the following settings: (1) source, 230°C; (2) transfer line, 280°C; (3) electron energy, 70 eV; and (4) solvent delay, 4 min.

**Phylogenetic analysis**

The phylogenetic analyses were conducted with Geneious software. MUSCLE 5.1 was used for multiple sequence alignments and FastTree 2.1.11 for tree construction. Microbial type I TCs were extracted from MARTS-DB^[4]^.

## References

[1] R. C. Edgar, *Nucleic. Acids. Res*. **2004**, *32*, 1792.

[2] K. F. Geoghegan, H. B. F. Dixon, P. J. Rosner, L. R. Hoth, A. J. Lanzetti, K. A. Borzilleri, E. S. Marr, L. H. Pezzullo, L. B. Martin, P. K. LeMotte, A. S. McColl, A. V. Kamath, J. G. Stroh, *Anal. Biochem.* **1999**, *267*, 169.

[3] A. B. Woodside, Z. Huang, C. D. Poulter, *Org. Synth.* **1988**, *66*, 211.

[4] M. Engst, M. Brokeš, T. Čalounová, A. Tajovská, M. Perković, R. Samusevich, R. Chatpatanasiri, T. Pluskal, **2025**, bioRxiv preprint, DOI: [10.1101/2025.05.11.653183](https://doi.org/10.1101/2025.05.11.653183).
